# Comprehensive evaluation of left ventricular deformation using speckle tracking echocardiography in normal children: comparison of three-dimensional and two-dimensional approaches

**DOI:** 10.1186/s12947-022-00273-6

**Published:** 2022-01-27

**Authors:** Doaa Aly, Nitin Madan, Laura Kuzava, Alison Samrany, Anitha Parthiban

**Affiliations:** 1 Ward Family Heart Center, Division of Cardiology, Children’s Mercy Hospital, UMKC School of Medicine, 2401 Gillham Road, Kansas, MO 64108 USA; 2 Texas Children’s Hospital, Baylor College of Medicine, Houston, TX USA

**Keywords:** Strain, Three-dimensional, Two-dimensional, Speckle tracking echocardiography, Reproducibility, Pediatric, Global, Regional, Left ventricle

## Abstract

**Background:**

Three-dimensional (3D) speckle tracking echocardiography (STE) can overcome some of the inherent limitations of two-dimensional (2D) STE; however, clinical experience is lacking. We aimed to assess and compare the feasibility, agreement, and reproducibility of left ventricular (LV) global longitudinal (GLS), and regional strain by 3D vs 2D STE in normal children.

**Methods:**

Healthy pediatric subjects (*n* = 105, age mean = 11.2 ± 5.5 years) were prospectively enrolled. Three-dimensional and 2D LV GLS, as well as regional strain in 16 myocardial segments were quantified. Bland Altman analysis, intra- class correlation coefficients (ICC), percent error and linear regression were used for agreement and correlation between the two techniques. Analysis and acquisition times were compared. Inter- and intra-observer reproducibility was assessed in 20 studies.

**Results:**

There was good to excellent agreement for 2D and 3D global longitudinal strain (ICC =0.82) and modest agreement for regional strain (ICC range 0.43–0.71). Both methods had high feasibility (88.6% for 2D vs 85.7% for 3D, *p* = 0.21), although 3D STE required significantly shorter acquisition and analysis time than 2D STE (acquisition time 1 ± 1.2 mins vs 2.4 ± 1 mins; *p* = 0.03, analysis time = 3.3 ± 1 mins vs 8.2 ± 2.5 mins; *p* = 0.001, respectively). Inter and intra-observer reproducibility was excellent for GLS by the two techniques (ICC = 0.78–0.93) but moderate to poor for regional strain (ICC = 0.21–0.64).

**Conclusion:**

Three-dimensional global LV strain is as feasible and reproducible as 2D strain, with good agreement yet significantly more efficient acquisition and analysis. Regional strain is less concordant and 2D and 3D values should not be used interchangeably. 3D LV GLS may represent a viable alternative in evaluation of LV deformation in pediatric subjects.

## Highlights

1. Adequate feasibility of 3D STE in pediatrics; comparable to 2D STE.

2. Significantly shorter acquisition and analysis time for 3D GLS compared to 2D GLS.

3. Excellent agreement between 3D and 2D LV GLS and moderate to poor agreement between regional strain values.

4. Excellent inter and intra-observer reproducibility for GLS by the two techniques, and fair to poor reproducibility for regional strain (higher for apical than basal regions).

## Introduction

Myocardial deformation assessed by two-dimensional (2D) speckle tracking echocardiography (STE) is a well validated quantitative marker of myocardial contractile function [1]. Two- dimensional STE has been widely reported in pediatric research for its ability to detect subclinical myocardial dysfunction in variety of pathologic conditions, but wide spread clinical implementation remains lagging [2–5]. This can be attributed to the time-consuming nature of data acquisition and analysis as well as the potential out-of-plane motion of speckles with subsequent inaccurate tracking. Three-dimensional (3D) STE has emerged as an attractive alternative technique that can overcome some of the inherent limitations of 2D STE. It can provide rapid and comprehensive deformation analysis from a single 3D full volume acquisition. This has resulted in a growing body of adult and pediatric literature illustrating the potential applications and clinical values of this modality [6–11].

While adult studies have demonstrated adequate feasibility, reproducibility, and accuracy of 3D STE- based global and regional strain analysis [12–14] similar large scale pediatric data are still lacking. This is particularly important as 3D STE has its own challenges in pediatric practice. Adequate 3D STE analysis generally requires a cooperative patient who can follow breath holding instructions for adequate ECG- gated multi-beat 3D acquisitions, which is often a limitation in children. There is also the challenge of the higher heart rate and potentially lower frame/volume rate to heart rate ratios in children compared to adults. Therefore, the aim of the present study was to assess and compare left ventricular (LV) global longitudinal strain (GLS) and regional strain analysis by 3D STE vs 2D STE in a large cohort of prospectively enrolled pediatric subjects.

## Methods

### Study participants and protocol

In this single center study, we prospectively recruited 105 consecutive pediatric subjects (age 0–18 years) with normal cardiac anatomy and function, who were referred to the Cardiology clinic at Children’s Mercy Hospital, Kansas City, for variety of reasons such as murmur, chest pain and palpitations. Subjects with echocardiographic evidence of congenital heart disease, cardiomyopathy, frequent ectopy hindering the evaluation of consecutive sinus beats, cardiac pacing or previous cardiac surgeries were excluded. Demographics and clinical information were obtained from the echocardiogram reports or the individual medical records. This study was approved by the Institutional Research Board of Children’s Mercy Hospital and informed assent/ consent was obtained from all subjects and their legal guardians.

### Data acquisition

Transthoracic echocardiography (TTE) was performed with the Philips ultrasound system “EPIQ 7”, using age and weight appropriate 2D and 3D matrix array transducers. Each subject underwent consecutive 2D and 3D TTE image acquisition by the same experienced operator using the same cardiac ultrasound system. Three-dimensional and 2D data sets were archived separately for blinded analysis.

For 2D echocardiography, three-beat clips of the multi-planar LV apical views (four, two and three chamber) were optimized for strain analysis using gain, compression, sector width and depth to maximize frame rate (> 60 frames/sec) and capture optimal myocardial tissue definition. Two-dimensional ejection fraction and end-diastolic volumes were calculated using the 5/6 area-length method [15].

For 3D echocardiography, full volume, multi-beat acquisitions of the LV were obtained from the apical, four chamber LV focused views. Image depth, width and gain were adjusted to achieve a volume rate of at least 30 volumes/sec. Acquisitions were optimized to include the entire LV including the apex and were obtained during breath hold (whenever possible) to avoid any stitch artifact.

### Strain analysis

**Fig. 1:**
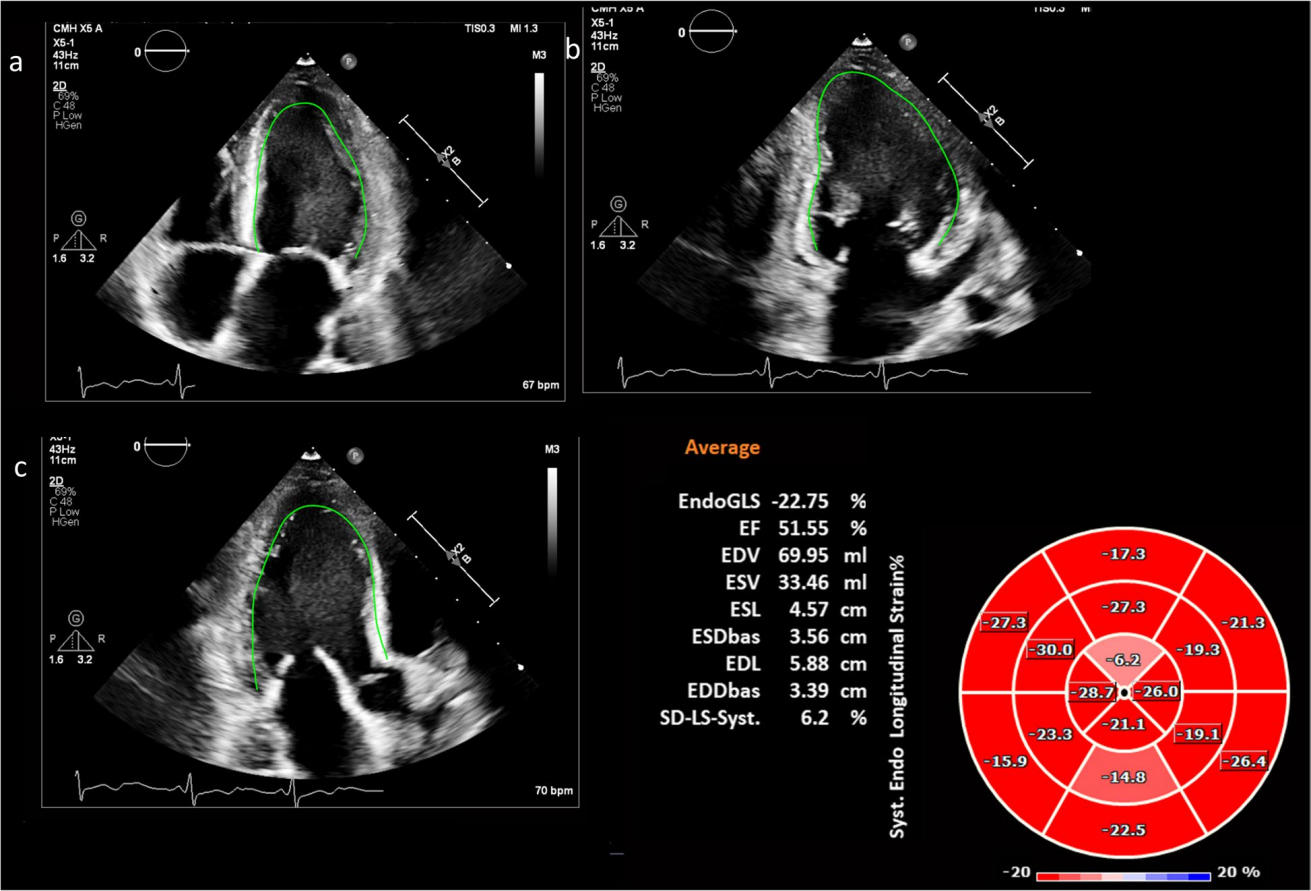
Left ventricular 2D GLS and regional strain. **a** LV four chamber view. **b** LV two chamber view. **c** LV three chamber view (**d**) Bull’s eye display of 2D peak regional strain. *LV,* left ventricle; *GLS,* global longitudinal strain

Two-dimensional and 3D echocardiographic acquisitions were analyzed using the TomTec software Image-Arena version 4.6 SP3, Unterschleissheim, Germany; 4D LV 3.1 for 3D STE and 2D CPA for 2D STE. For 2D GLS analysis, a single cardiac beat with the best endocardial definition was used. The region of interest was traced in end-systole by delineating the endomyocardial contour from medial to lateral mitral valve (MV). Tracking was automatically performed and manually adjusted if necessary, based on visual inspection of tracking (Fig. 1). As per EACVI task force recommendations [16], GLS was calculated as the change in the “length of the line” in each apical view and LV GLS was calculated as the average of GLS of the three LV apical views.

**Fig. 2:**
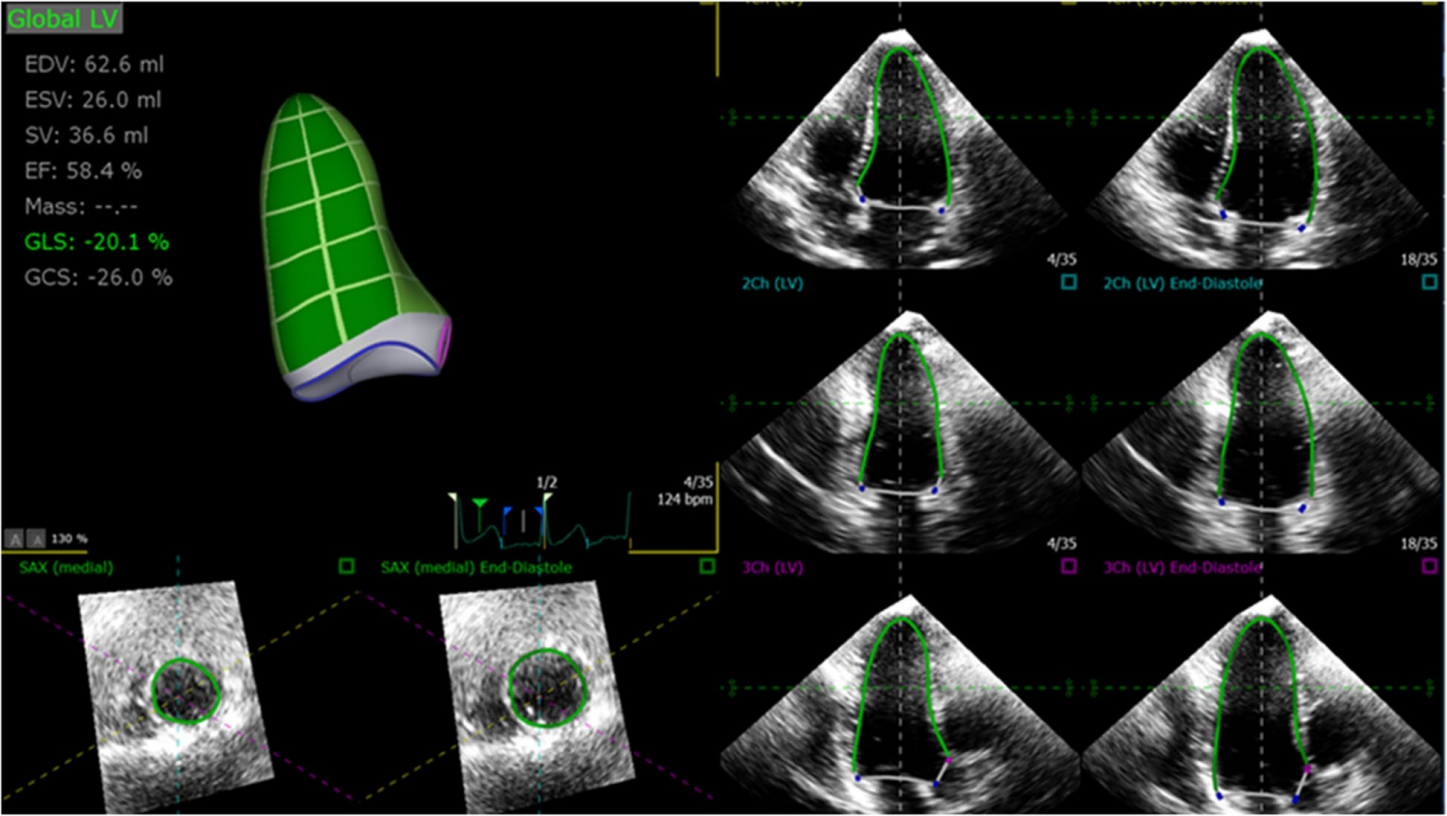
Left ventricular 3D GLS. Automated LV endocardial tracking in the four, three and two chambers (**b**) Bull’s eye display of 3D peak regional strain. *LV*, left ventricle; *GLS,* global longitudinal strain

For 3D analysis, full-volume 3D data sets with minimal amount of dropout and stich artifacts were analyzed on a separate computer workstation by a separate cardiologist to minimize bias. Three-dimensional LV volumes, LVEF and strain were measured using the semi-automated border detection algorithm for four-, two-, and three-chamber views (Fig. 2). After confirming the anatomic landmarks (mitral annulus and LV apex), the software generated end-diastolic and end-systolic endocardial contours of the LV in each of the above cut-planes. These contours were manually adjusted when necessary, to optimize boundary position and tracking throughout the cardiac cycle (Fig. 2). The LV outflow tract, papillary muscles, and trabeculations were included within the LV cavity.

**Fig. 3:**
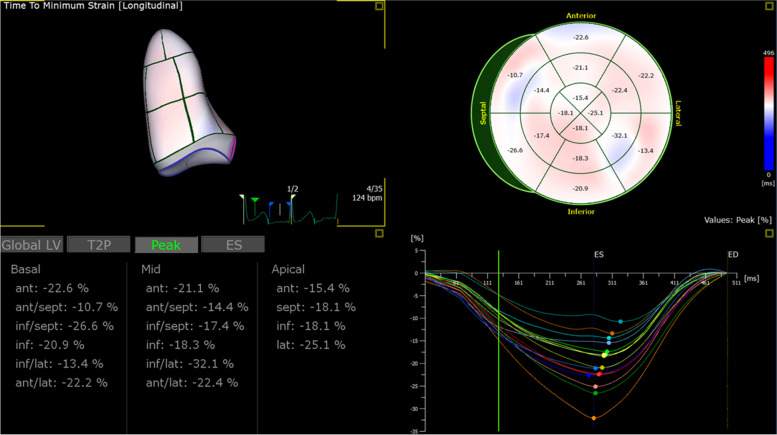
Left ventricular 3D regional strain. Bull’s eye display of 3D peak regional strain. *LV*, left ventricle; *ant*, anterior; *sept,* septal; *inf,* inferior; *lat,* lateral

For regional strain analysis, 2D and 3D images of the LV wall were automatically divided into 16 segments and displayed as a bull’s eye figure. Two-dimensional and 3D strain analysis were performed by independent blinded readers (Figs. 1 and 3).

### Reproducibility

Strain analysis was repeated on 20 randomly selected patients by the same reader after 4 weeks for intra-observer variability and by a second reader for inter-observer variability.

### Feasibility and efficiency

Patients with poor quality 2D images defined as those with foreshortened apical views, significant lung artifacts obscuring the LV apex or LV walls, and or frame rate < 60 frame per second) were excluded from analysis. Poor quality 3D full volume acquisitions defined as those with a significant stitch artifact, poorly visualized MV, or apex, and or volume rate < 30 volumes per second) were also excluded. For both 2D and 3D strain analysis, tracking was automatically performed by the strain analysis software, however manual edits were made as deemed necessary and analysis was accepted only after visual inspection. If tracking was suboptimal, the endocardial border was retraced. If satisfactory tracking was not accomplished after three re-tracings, specifically, if more than three segments had poor tracking, the study was deemed not analyzable and was subsequently excluded. Time required for acquisition and for offline analysis was recorded for the 2 modalities.

### Statistical analysis

Statistical analysis was performed using SPSS for Windows version 26.0 (SPSS, Inc., Chicago, IL, USA). Kolmogorov-Smirnov test was used to test for normality. Categorical data were presented as percentages, continuous and normally distributed data were presented as mean ± SD, while data deviating from normality were expressed as median and inter-quartile range. Correlation between 2D and 3D strain was performed using Pearson correlation coefficient (*r*) and linear regression. As recently recommended by Bunting et al. [17], we used Bland Altman analysis including bias and limits of agreement, intra- class correlation coefficients (ICC); (two-way mixed model, absolute consistency between single measurements) and percent error (100 x difference/mean) to quantify the agreement between the 2 techniques.

Good agreement was indicated by near zero bias and narrow limit of agreement (LOA) relative to the measured values. Guidelines recently recommended by Bunting et al. [17] were used for the interpretation of ICCs. An ICC < 0.40 denoted poor agreement, 0.40–0.59 was fair, 0.60–0.74 was good, and ICC of 0.75–1.00 indicated excellent agreement. Statistical significance was indicated by *P* value < 0.05.

## Results

### Demographic and echocardiographic characteristics

Baseline demographic and echocardiographic characteristics are summarized in Table 1. The mean age of subjects included was 11.2 ± 5.5 years, 20 of whom were ≤ 5 years old. All subjects had normal LV systolic function as quantified by the 5/6 area-length 2D LV ejection fraction (EF) with a mean of 62 ± 4.1% and by 3D EF with a mean of 61 ± 3.6%.

**Table 1:** Baseline demographic and echocardiographic characteristics

Variables	value
Males	57%
Age (y)	11.2 ± 5.5
Body surface area (m^2^)	0.4 ± 0.6
Heart rate (beats/min)	87 ±30
2D LV EF (%)	62 ± 4.1
3D LV EF (%)	61 ± 3.6
2D LV EDV Z score	1.3 ± 0.3
3D LV EDVi (ml/m^2)^	70.4 ±8.1

Data are expressed as mean ± SD (range) or as percentages

*Y* years, *min* minutes, *LV* left ventricle, *EF* ejection fraction, *EDV* end diastolic volume, *EDVi* end diastolic volume indexed to body surface area

### Feasibility and efficiency

One hundred five subjects were originally enrolled in the study. Eight subjects were excluded from 2D analysis due to poor quality 2D images. Four additional 2D datasets were excluded due to poor tracking upon strain analysis, with an overall 2D STE feasibility rate of 88.6%. Fifteen subjects were excluded from 3D analysis due to poor quality 3D full volume acquisitions and inadequate tracking, (nine of whom were younger than 5 years old, with heart rate > 120 bpm) with an overall 3D STE feasibility rate of 85.7%. This had a statistically insignificant *p* value when compared to 2D STE feasibility rate (85.7 vs 88.6%, *p* = 0.21).

The average temporal resolution of the 2D and 3D datasets included was 78 ± 9 frames per second, and 39 ± 7 volumes per second, respectively. The average acquisition time and average offline analysis time was significantly shorter for 3D STE vs 2D STE (acquisition time = 1 ± 1.2 mins vs 2.4 ± 1 mins; *p* = 0.03, analysis time = 3.3 ± 1 vs 8.2 ± 2.5 mins; *p* = 0.001, respectively).

### Inter-technique agreement and correlations

**Fig. 4:**
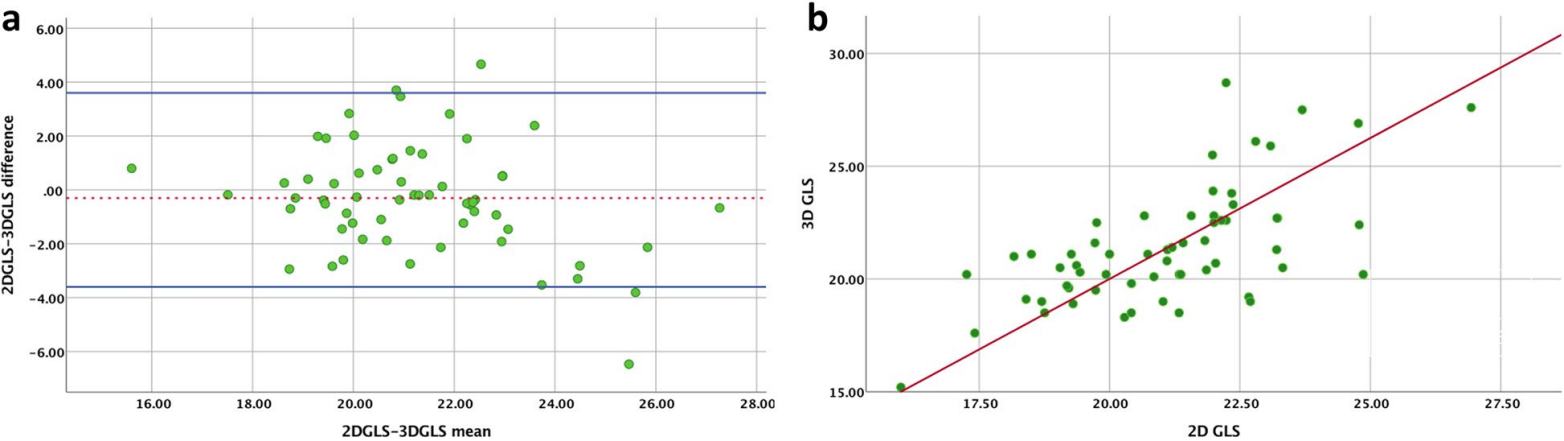
Inter-technique agreement and correlation between 2D and 3D LV GLS. **a** Bland-Altman plot illustrating the bias (mean difference) and limits of agreement between 2D GLS and 3D GLS. **b** Scatter plot illustrating the linear correlation between 2D and 3D GLS*. LV*, left ventricle; *GLS,* global longitudinal strain

Comparison of the global and regional strain values by 2D and 3D STE for the entire cohort is presented in Table 2. The correlation and measures of inter-technique agreement between 2D and 3D strain measurements are presented in Table 3**.** There was an insignificant difference, strong and significant correlation (*r* = 0.73, *p* < 0.05) as well as excellent agreement (Bias = − 0.3, LOA = − 3.7 -3.6, percent error = 1.4% and ICC = 0.82) between 2D and 3D GLS (Fig. 4). On the other hand, there was a varying degree of correlation (*r* = 0.21–0.51) and agreement between the different myocardial segments for regional strain (Bias = − 0.2- 3.7, LOA = − 21.7- 19.7, percent error = 2.2–30.3% and ICC = 0.37–0.74) with overall weaker correlation and less agreement in the basal than apical segments (Table 2).

**Table 2:** Absolute strain values and % performed of 2D and 3D global and regional strain

Variables	2D STE		3D STE	
	Mean ± SD	%performed	Mean ± SD	%performed
**GLS**	−21.2 ± 2.4	89	− 20 ± 2.1	87
**Basal ante septum**	− 17.7 ± 7.3*	86	− 25.9 ± 7.7*	83
**Basal anterior**	− 16.37 ± 5.7	86	− 20.7 ± 6.3*	85
**Basal lateral**	− 18.5 ± 6.1	89	− 21.1 ± 5.2*	86
**Basal posterior**	−21.7 ± 5.9	86	− 25 ± 7.2*	87
**Basal inferior**	− 23.5 ± 7.6	89	− 18.5 ± 3.7 *	86
**Basal septum**	−14.5 ± 5.5	89	− 19.7 ± 4.9*	86
**Mid ante septum**	− 18.9 ± 6.8	86	−18.8 ± 7.6	87
**Mid anterior**	− 17.2 ± 5.6	89	−21.1 ± 4.5*	86
**Mid lateral**	− 19.7 ± 5.8	89	− 21.8 ± 5.6*	85
**Mid posterior**	− 19.3 ± 7.2	86	−19.2 ± 5.3	87
**Mid inferior**	−19.6 ± 6.0	85	− 19 ± 7.6	87
**Mid septum**	− 18.7 ± 5.4	84	−19.3 ± 7.0	86
**Apical anterior**	−24.1 ± 4.8	85	−22.8 ± 5.4*	85
**Apical lateral**	−23.1 ± 5.1	84	− 24.3 ± 6	83
**Apical inferior**	− 18.7 ± 6.6	85	− 17.7 ± 6.5	87
**Apical septum**	− 16.8 ± 6.2	89	−18.8 ± 6.2*	87

*GLS* global longitudinal strain, *ante* anterior, *STE* speckle tracking echocardiography, *SD* standard deviation. * indicates *p* < 0.05

**Table 3:** Inter- technique agreement of 2D and 3D global and regional strain values

Variables	Bland Altman		% error	ICC		Correlation
	Bias	LOA	Mean	Mean	95% CI	***R***
**GLS**	−0.3	3.6 to − 3.7	1.4	0.82	0.61–0.87	0.73*
**Basal ante septum**	−6.5	8.7 to − 21.7	30.3	0.43	−0.23 - 0.71	0.32
**Basal anterior**	−4.5	10.1 to −19	25.4	0.41	−0.26 - 0.62	0.21
**Basal lateral**	−2.8	8 to − 13.6	14.4	0.49	0.40–0.47	0.51
**Basal posterior**	−3.1	9.9 to −15.9	13.4	0.48	0.25–0.53	0.34
**Basal inferior**	3.7	19.7to −12.3	17.2	0.42	0.27–0.61	0.32
**Basal septum**	−5.7	5.4 to −17.4	34.2	0.49	0.23–0.54	0.29
**Mid ante septum**	−0.2	13.3 to −12.7	1.3.5	0.74	0.47–0.81	0.51*
**Mid anterior**	−2.3	9 to −13.5	12.3	0.42	−0.32 - 0.61	0.22
**Mid lateral**	−1.7	12.2to − 15.7	8.2	0.42	−0.28 - 0.56	0.24
**Mid posterior**	0.6	13.6 to-12.1	3.1	0.53	0.37–0.65	0.41*
**Mid inferior**	−1.4	13.5 to-16	1.9	0.37	−0.28 - 0.51	0.30
**Mid septum**	−1.2	11.5 to-13.9	2.2	0.51	0.39–0.70	0.40
**Apical anterior**	0.9	11.9 to-10	4.1	0.50	0.34–0.71	0.32
**Apical lateral**	−1.5	9 to − 12	6.3	0.48	0.36–0.65	0.29
**Apical inferior**	−1.2	13.6 to −15.6	5.1	0.49	0.30–0.66	0.43*
**Apical septum**	−1.1	11.1 to −13.1	6.4	0.63	0.42–0.83	0.41*

*GLS* global longitudinal strain, *ante* anterior, *STE* speckle tracking echocardiography, *LOA* limit of agreement (95% confidence interval, *ICC* intra-class coefficient, *CI* confidence interval, *R* Pearson’s correlation coefficient. % error = 100 X (difference/ mean). * *P* < 0.05

### Reproducibility

Table 4 displays the degree of intra and inter-observer reproducibility (measured by ICC) for 2D and 3D STE global and regional strain. The intra and inter-observer reproducibility was equally robust for LV GLS between the two techniques (ICC range of 0.81–0.92). There was less degree of reproducibility among the different myocardial segments (ICC range of 0.21–0.73), than with global strain, with a trend towards higher ICCs among the apical than the basal regions. This did not seem to vary between 2D and 3D STE techniques.

**Table 4:** Inter and intra- observer reproducibility (expressed as ICC) of 2D and 3D global and regional strain values

Variables	Intra-observer reproducibility		Inter-observer reproducibility	
	2D STE	3D STE	2D STE	3D STE
**GLS**	0.92	0.87	0.88	0.81
**Basal ante septum**	0.32	0.43	0.23	0.33
**Basal anterior**	0.41	0.37	0.27	0.31
**Basal lateral**	0.21	0.34	0.21	0.41
**Basal posterior**	0.33	0.41	0.27	0.32
**Basal inferior**	0.35	0.31	0.29	0.42
**Basal septum**	0.43	0.43	0.31	0.38
**Mid ante septum**	0.51	0.56	0.47	0.61
**Mid anterior**	0.47	0.51	0.39	0.37
**Mid lateral**	0.43	0.47	0.31	0.51
**Mid posterior**	0.31	0.41	0.27	0.46
**Mid inferior**	0.44	0.52	0.28	0.31
**Mid septum**	0.57	0.61	0.41	0.52
**Apical anterior**	0.72	0.68	0.72	0.66
**Apical lateral**	0.73	0.76	0.63	0.67
**Apical inferior**	0.55	0.60	0.31	0.42
**Apical septum**	0.43	0.51	0.37	0.46

*ICC* intra-class coefficient, *GLS* global longitudinal strain, *ante* anterior, *STE* speckle tracking echocardiography

## Discussion

Three-dimensional STE is a new imaging technique designed for myocardial deformation analysis from 3D data sets, with the potential to overcome some of the inherent limitations of 2D STE. To our knowledge, this is the first pediatric study to provide comprehensive, head-to-head comparison of LV 3D and 2D STE analysis in a large cohort of healthy children.

Our study demonstrated the following: 2- Good feasibility of 3D STE, that is comparable to 2D STE (85.7 vs 88.6%, *p =* 0.21). 2- Significantly shorter acquisition and analysis time for 3D STE vs 2D STE (acquisition time = 1 ± 1.2 mins vs 2.4 ± 1 mins; *p =* 0.03, analysis time = 3.3 ± 1 vs 8.2 ± 2.5 mins; *p* < 0.001, respectively). 3- Excellent agreement between 3D and 2D GLS (ICC = 0.82 (0.61–0.87)), and varying agreement between regional strain values (ICC = 0.21–0.73); higher for the apical than the basal segments. 4- Equally robust inter and intra-observer reproducibility for GLS by the two techniques (ICC range of 0.81–0.92), and fair to poor 2D and 3D reproducibility for regional strain (higher for apical than basal segments), with an ICC range of 0.21–0.73.

### Speckle tracking echocardiography for assessment of LV mechanics

Two-dimensional STE is a technique that utilizes frame-by-frame tracking of acoustic speckles to provide a quantitative assessment of myocardial deformation [2]. Since, there are no geometric assumptions or significant influence of loading conditions, this technique is believed to be superior to conventional 2D echocardiographic measures of ventricular function such as EF. However, given the complex spatial arrangement of the LV myofibrils and simultaneous multi-vector contractions, it is not unexpected for strain measurements limited to three one-dimensional planes to suffer planar simplification and out-of-plane motions.

To circumvent this limitation, 3D STE has emerged as a newer, potentially more physiologically sound technique to quantify ventricular deformation, which by nature is a 3D phenomenon. Therefore, 3D STE is not affected by the out- of- plane or twisting motion or apical foreshortening. Additionally, and in contrast to 2D STE, a single apical full volume acquisition can provide comprehensive data about 3D LV global and regional strain, EF, volumes, and sphericity index. Moreover, 3D STE can provide novel myocardial deformation parameters such as area strain [18], principal strain and tangential strain [19].

While 3DSTE has the potential to be established as the new gold-standard for assessing LV function, it is not without limitations. As with 3D echocardiography in general, 3D strain may suffer from lower temporal and spatial resolution, which may interfere with adequate tracking. Additionally, multi-beat acquisition without significant stitching artifact between sub-volumes requires a significant degree of cooperation on behalf of the patient in order to follow breath holding instructions- particularly a challenge in pediatrics. Specialized transducers and software as well as sonographer training with an initial steep initial learning curve are required. Our study is the first prospective study to explore the applicability of 3D STE in the normal pediatric population, by comparing its feasibility and reproducibility to the more established 2D STE*.*

### Inter-technique agreement and correlation of global strain

Akin to adult reports, our study noted good agreement and strong correlation between 2D and 3D LV GLS with overall slightly lower 3D GLS values. A recent meta-analysis performed to compare global 2D and 3D strain values in adults, with 3846 paired comparisons of GLS included from 36 publications, demonstrated insignificant difference when TomTec software was used [19]. Interestingly, the pooled mean values of 3D GLS for other vendors such as GE and Toshiba were significantly lower than that of 2D GLS. While on the pediatric side, we are only aware of a single study where the two techniques were compared. In this study, analysis was performed using an older version of TomTec, in a smaller, mixed cohort of patients with congenital heart disease wherein the ventricular geometry was likely more abnormal and varied than in our study [20]. Nevertheless, strong correlation (*r* = 0.92 *p* < 0.001) and minimal difference were still appreciated between 2D and 3D analyis, although (and unlike our results) 3D GLS tended to have higher mean values than 2D GLS.

The variability between 2D and 3D strain analysis could be due to number of factors, such as the discordant frame/volume rates between the two techniques. The two techniques also have different tracking and analysis algorithms with 3D strain being calculated from cubes with specific 3D patterns of acoustic markers (block matching) vs 2D strain which is calculated based on areas that contain specific natural acoustic markers, frame by frame, within the region of interest (pattern matching) [13].

### Inter-technique agreement and correlation of regional strain

3D STE is capable of providing quantitative regional strain analysis. The role of 3D regional strain is still evolving, with studies demonstrating useful applications in the assessment of myocardial ischemia and viability, although reliable measurement of regional strain remains challenging [19, 21, 22]. In our study, there was a varying degree of correlation and agreement between 2D and 3D peak longitudinal strain values among different myocardial segments. Correlation was weaker, and limits of agreements were wider in the basal compared to apical segments, which is likely since basal segments are farther in the field and have the most active excursion, hence are the hardest to track by 2D and 3D STE.

The overall modest agreement and correlation between 2D and 3D regional strain is consistent with the currently available clinical adult studies [23]. This is thought to be secondary to difficult matching of corresponding LV segments between 2D and 3D STE. Regional strain also tends to be significantly more sensitive to noise and measurement error than global strain as it does not benefit from the favorable influences of averaging [24]. Therefore, individual 2D and 3D regional strain values may not be interchangeable. Instead, grouped segments (for instance by coronary territories), might suffer less from the aforementioned challenges and potentially provide better agreement and more clinically relevant data; an approach that we intend to investigate in the future.

### Feasibility and efficiency

In this study, we report a feasibility of 88.6% for 2D STE and 85.7% for 3D STE. While our 2D STE feasibility data seem to be in good match with the previously published pediatric and adult data (average 80–97%) [13, 25, 26], our 3D STE feasibility rates seem to be significantly higher than the average reported rates (average 63–83%) [19, 27, 28].

The accuracy of 3D STE largely depends on optimal image quality with sufficient frame rate which require a remarkable amount of dedicated training and practice to obtain. In our echocardiography lab, we utilize 3D imaging on a regular basis and are currently reporting 3D LV EF on all our standard function-focused pediatric echocardiograms. For this goal to be finally achieved, our sonographers received extensive one-on-one training and have consequently developed the skills needed for optimal 3D volumetric imaging. Our results confirm that with adequate training, 3D strain analysis can be nearly as feasible as 2D in the pediatric population [26, 29–31].

Our results also demonstrate a significantly higher efficiency with significantly shorter acquisition and analysis time for 3D vs 2D STE. This is somewhat expected given the fact that a single 3D apical dataset is required for both 3D GLS, while 3 different apical planes need to be optimized and acquired for 2D GLS. This is of utmost importance, as high-volume echocardiography laboratories strive for an accurate, reproducible as well as efficient quantitative method of ventricular function to adopt into clinical routine on a wider scale.

Reproducibility of 3D STE has been reported as acceptable to excellent in several studies with intra-observer variability ranges from 1 to 13% and inter-observer variability ranges from 2 to 14% [12, 32, 33]. As with 2D STE, our data showed higher variability among 3D regional than LVGLS. The excellent reproducibility of global data observed in our study could be reflective of the role of semiautomatic/ automatic tracking and segmentation in minimizing variability during strain analysis.

## Limitations

We consider the following potential limitations of our study: 1- Our study cannot establish superiority of one technique over the other, since there is no true gold standard method to measure myocardial deformation for comparison currently. Our study on the other hand, could act as a precursor to large single or multicenter studies to validate both techniques against a gold standard for functional imaging such as CMR and/ or invasive measures of cardiac function. 2- Despite the fact that we had a large cohort of subjects in our study, our sample size could potentially benefit from a larger group of volunteers with congenital or acquired heart disease. 3- While a previous study we published recently did show good intervendor agreement of 2D GLS [25], to our knowledge, there are no current similar data available about the intervendor variability of 3D strain in pediatrics. Therefore, our results should be interpreted with caution when different echo machines/ post processing analysis platforms are being used.

## Conclusions

We have demonstrated that pediatric LV 3D STE systolic strain analysis is comparable to 2D STE with regards to feasibility and reproducibility while providing much faster acquisition and analysis than by 2D STE. Since 3DE allows for simultaneous assessment of LV volumes, EF and multidirectional components of strain, there are distinct advantages to workflow in clinical practice and could be considered in lieu of 2D STE particularly for LV GLS. Given the variability between the two techniques for regional strain, we do not believe 2D and 3D regional strain values should be used interchangeably.

## Data Availability

The data that support the findings of this study are available from the corresponding author upon reasonable request.

## Abbreviations

Ante: Anterior
CI: Confidence interval
EF: Ejection fraction
EDV: End diastolic volume
EDVi: End diastolic volume indexed to body surface area
GLS: Global longitudinal strain
ICC: Intra-class correlation coefficients
LOA: Limits of agreement
LV: Left ventricular
MV: Mitral valve
*r*: Pearson correlation coefficient
STE: Speckle tracking echocardiography
TTE: Transthoracic echocardiograms
2D: Two-dimensional
3D: Three-dimensional
